# Corrigendum to “Role of Interleukin-18 in Modulation of Oral Carcinoma Cell Proliferation”

**DOI:** 10.1155/2018/9731639

**Published:** 2018-10-17

**Authors:** Mediators of Inflammation

In the article titled “Role of Interleukin-18 in Modulation of Oral Carcinoma Cell Proliferation” [1], KB cells have been found to be cross-contaminated by HeLa cells [2], which means that KB is really a cervical cancer line and not an oral cancer line. Accordingly, no conclusions can be drawn relating specifically to oral cancer.

The authors could not be contacted.
